# Crosstalk between autophagy and ferroptosis in diabetes

**DOI:** 10.1016/j.gendis.2025.101936

**Published:** 2025-11-10

**Authors:** Erlian Xie, Xuerong Wei, Zijun Zheng, Qiuyi Yu, Mengqian Liu, Huihui Zhang, Ziwei Jiang, Yanbin Gao, Lei Yang

**Affiliations:** Department of Burns, Nanfang Hospital, Southern Medical University, Guangzhou, Guangdong 510515, China

**Keywords:** Autophagy, Co-mechanism, Diabetes mellitus, Diabetic complications, Ferroptosis

## Abstract

Diabetes is a multifactorial metabolic disease involving complex disruptions in cellular homeostasis and multiple forms of regulated cell death. Among them, the interaction between autophagy and ferroptosis has recently gained increasing attention. Autophagy is a catabolic process essential for degrading damaged organelles and misfolded proteins, thus preserving cellular integrity. Ferroptosis, on the other hand, is a newly identified, iron-dependent form of cell death characterized by excessive lipid peroxidation. Emerging evidence suggests that these two processes are intricately linked through shared regulatory pathways involving iron metabolism, lipid homeostasis, and the antioxidant system. Their crosstalk plays crucial roles in key diabetic pathologies, including pancreatic β-cell dysfunction, insulin resistance, and vascular complications. This review provides a comprehensive overview of the molecular mechanisms underlying autophagy–ferroptosis interactions in diabetes and highlights how their cooperative or antagonistic actions contribute to disease progression. Additionally, we discuss novel therapeutic strategies aimed at modulating this interplay, which may offer promising avenues for improving outcomes in diabetes and its complications. Further studies are needed to define precise molecular targets and facilitate clinical translation.

## Introduction

**Figure 1:**
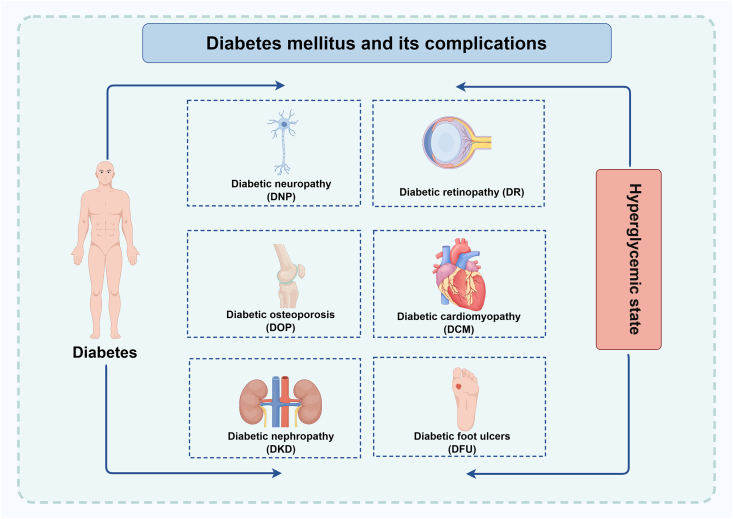
Diabetes mellitus and its various complications. Long-term hyperglycemic environments ultimately lead to a range of severe complications, such as diabetic foot ulcers, diabetic nephropathy, diabetic osteoporosis, diabetic neuropathy, diabetic retinopathy, and diabetic cardiomyopathy. The figure was generated with Figure Draw.

Type 1 diabetes mellitus and type 2 diabetes mellitus are the two main kinds of diabetes, which are both among the most prevalent and dangerous chronic diseases in existence today.^1^^,^^2^ The number of diabetics worldwide is predicted to reach 537 million by 2021, and this number is projected to climb by 46% by 2045, demonstrating a rapid and ongoing rise in diabetes prevalence.^1^ Microvascular and macrovascular lesions can result from long-term glucose metabolism problems, microangiopathies, neurotrophic factor deficiency, and oxidative stress-induced damage. These conditions can cause blood vessel damage and raise the risk of complications like diabetic nephropathy, diabetic retinopathy, and diabetic cardiomyopathy. Nevertheless, elevated blood sugar levels can also harm the neurological system, resulting in neuropathy and neuropathic illness (diabetic neuropathy). Furthermore, elevated blood sugar levels have been linked to immune system dysfunction and an increased risk of diabetic foot ulcers^3^ (Fig. 1). Epidemiological investigations have revealed that diabetic cardiomyopathy triples the risk of heart failure, while diabetic nephropathy has emerged as the predominant etiology of end-stage renal disease. Notably, diabetic retinopathy constitutes the leading cause of adult-onset blindness. According to the statistics by the International Diabetes Federation, cardiovascular complications account for approximately 70% of diabetes-related mortality, with their substantial disability and mortality rates profoundly impairing patients' quality of life and imposing overwhelming burdens on healthcare systems.^4^ Consequently, elucidating the molecular pathogenesis of diabetic complications and developing targeted therapeutic interventions represent critical unmet clinical needs. Many studies argue that the disease’s underlying causes encompass genetic inheritance, irregular dietary patterns, obesity, lifestyle factors, and occupational influences.^5^ Its heterogeneous etiological pathology involves insulin intolerance, beta cell dysfunction, or a concoction of both, along with disorders in carbohydrate, protein, and lipid metabolism.^6^ The identification of mechanisms of diabetes mellitus will provide us with the opportunity to develop rational therapeutic regimens to improve clinical outcomes. Recent studies have proved that ferroptosis and autophagy are associated with the regulation of diabetes mellitus and that autophagy plays an important role in inducing ferroptosis.

In contrast to apoptosis, cuproptosis, autophagy, necroptosis, and pyroptosis, ferroptosis is a regulated form of cell death mediated by iron-dependent accumulation of lipid peroxides.^7^^,^^8^ It largely results from an imbalance between lipid peroxide production and clearance, which plays a major role in the development of many metabolic illnesses. For example, ferroptosis appears to be a key factor in diabetes, with altered ferroptosis observed in various diabetic complications such as the heart, kidneys, blood vessels, and nerve tissue.^9^, ^10^, ^11^, ^12^, ^13^, ^14^, ^15^, ^16^, ^17^, ^18^, ^19^, ^20^, ^21^ Therefore, a viable approach to treating diabetes and its complications may involve understanding the regulatory mechanisms causing ferroptosis and creating specific therapeutic interventions or medications against this process.

Autophagy, a highly conserved process found in all eukaryotic cells, serves as a fundamental mechanism of “self-digestion”. It encompasses processes such as degradation, recycling, removal, and synthesis of new proteins, playing a crucial role in various aspects of cellular life, including energy metabolism, signal transduction, nutrition, and immune responses. To maintain the normal structure of islets, the crucial process of autophagy is indispensable. Nevertheless, autophagy is suppressed in a high-glucose environment, leading to problems, insulin resistance, and compromised islet function.^22^ In recent years, the study of diabetes prevention and therapy has increasingly focused on the connection between autophagy and problems from diabetes. With greater knowledge of the mechanisms behind diabetes and associated consequences, driven by advances in cell biology, proteomics, molecular biology, and metabolomics, the role of autophagy in diabetes has been elucidated.^23^

Recent research indicates that autophagy significantly contributes to ferroptosis induction, particularly in the context of diabetes and its complications. Unveiling the interplay between ferroptosis and autophagy not only enhances our mechanistic comprehension of these two forms of programmed cell death but also unveils novel therapeutic avenues for managing diabetes. This review offers a current perspective on the mechanisms underlying ferroptosis and autophagy, elucidating potential pathways or compounds involved in their interplay. Subsequently, we explore the prospective therapeutic implications of targeting this interaction in the context of diabetes and its complications, presenting it as a promising therapeutic avenue for future interventions.

## The introduction of ferroptosis and autophagy

### Ferroptosis

Ferroptosis, a new type of non-apoptotic cell death, is mainly characterized by increased lipid reactive oxygen species (ROS) and intracellular accumulation of ferrous ions, with important implications for cellular metabolism. Research indicates that the occurrence of ferroptosis can be regulated by multiple metabolic pathways. The mechanisms that induce ferroptosis are often associated with the pathways that generate lipid peroxides, such as iron metabolism and lipid peroxidation catalyzed by fatty acid enzymes. The pathways that inhibit ferroptosis, on the other hand, involve antioxidant systems that scavenge excessive lipid peroxides, including systems like the Xc^–^ system-GSH-GPX4, the NAD(P)H/FSP1/CoQ10 system, and the GCH1/BH4/DHFR system.

It is well-known that ferroptosis is most significantly affected by iron, a trace metal that is vital to human health and has enzymatic activity. It has been shown that ferroptosis can be promoted or inhibited by modulating the expression and function of many proteins and genes involved in iron metabolism. For example, increasing the expression of nuclear receptor coactivator 4 (NCOA4) in cells can mediate the autophagic turnover of ferritin heavy chain 1 under iron deficiency conditions.^24^ Ferritin-mediated iron transport may be hampered by the depletion of NCOA4 and poly(rC)-binding protein 1 (PCBP1).^25^ Furthermore, PCBP1 inhibits the dual function of Beclin1 and Arachidonic acid 15-lipoxygenase mRNA to help reduce cellular susceptibility to ferroptosis.^26^ More and more studies have found that proteins such as heat shock protein β, ferri-responsive element binding protein 2, transferrin receptor, and divalent metal transporter 1 (DMT1) can regulate ferroptosis by regulating iron input, output, or storage.^27^, ^28^, ^29^

Apart from the primary mechanisms of ferroptosis elucidated above, there exist additional pathways of ferroptosis that operate independently of iron metabolism. It has been discovered that iron can readily catalyze the conversion of polyunsaturated fatty acids, one of the byproducts of glucose metabolism, to lipid peroxides through the Fenton reaction. This process results in ferroptosis, which ultimately compromises the integrity of the cell membrane and impairs membrane function.^30^ Long-chain acyl-CoA synthetase (ACSL4) and lysophosphatidylcholine acyltransferase 2 are the primary enzymes involved in this process that facilitate the development of lipid peroxidation. By a variety of pathological mechanisms, ferroptosis may be facilitated by upregulating the expression or activity of both.^31^^,^^32^

System Xc^–^ is a glutamate/glutathione countertransport system consisting of two separate proteins, solute carrier family 7 member 11 (SLC7A11) and solute carrier family 3 member 2, which are mainly involved in cystine uptake and transport. Glutathione peroxidase 4 (GPX4) is a central regulator of ferroptosis whose anti-ferroptosis activity depends on the catalytic function of its selenocysteine residue and requires the participation of glutathione (GSH). GSH/GSSG is an important intracellular antioxidant system that converts reduced GSH to oxidized GSH, a process that converts reactive PLOOH to inactive PLOH to inhibit lipid peroxidation. Therefore, reducing cystine uptake and transport, and inhibiting or blocking GSH/GSSG conversion, decreases GPX4 synthesis and lipid repair function.^33^ For instance, the expression level of SLC7A11 is usually positively correlated with the activity of reverse transporter proteins; p53 was found to inhibit SLC7A11, reducing glutathione synthesis and thus GPX4 activation.^34^ The well-known inducer RSL3 directly inactivates GPX4, whereas Erastin induces ferroptosis by indirectly inactivating GPX4 mainly by inhibiting cystine entry. More recent studies have also identified Fin56 as a novel ferroptosis inducer, which triggers ferroptosis by promoting GPX4 protein degradation through a pathway that is not yet fully understood. Since then, GPX4 has been shown to abrogate lipid peroxidation and protect cells against iron metastasis.^35^^,^^36^

The NAD(P)H/FSP1/CoQ10 system represents an alternative antioxidant system that is independent of GPX4. In the absence of GPX4, ferroptosis suppressor protein 1 (FSP1) utilizes NAD(P)H to regenerate ubiquinol from its oxidized form, ubiquinone, thereby inhibiting phospholipid peroxidation in cells.^37^ Additionally, it can synergize with non-canonical vitamin K redox cycling^38^ and other mechanisms to jointly suppress lipid peroxidation and the occurrence of ferroptosis, thereby protecting cells from damage.

Lastly, another antioxidant system is the GCH1/BH4/DHFR. Tetrahydrobiopterin (BH4) is an antioxidant that captures free radicals and serves as an essential cofactor for all nitric oxide synthases. Insufficient availability of BH4 can lead to decoupling of nitric oxide synthases and the production of highly oxidative radicals.^39^ By synthesizing and providing BH4 as an antioxidant, this system scavenges ROS and lipid peroxides within cells, thereby inhibiting the occurrence of ferroptosis.^40^

### Autophagy

Autophagy is an important intracellular degradation and recycling mechanism. It is a protective stress response mechanism that regulates various metabolic pathways to achieve cell self-renewal and maintain cell steady state and function.^41^ Autophagy is divided into three types: macroautophagy, microautophagy, and chaperone-mediated autophagy, of which macroautophagy is the most common type. The process of autophagy involves several stages, including the initial stage of autophagy, isolated membrane and autophagosome formation, autophagosome-lysosome fusion stage, and lysis of autophagosomes.^42^ Numerous pathways and intricate mechanisms govern autophagy, and more than 40 autophagy-related genes (ATGs) have been found to participate in the execution of autophagy.^43^

In 2004, Yu-Yun Chang et al discovered that ATG17 kinase, which forms a complex of ATG1 and ATG13, is a key regulator of autophagy initiation,^44^ and in the following decade, a new phenomenon was found that promotes autophagy, that is, AMP-activated protein kinase (AMPK) directly activates ULK1 in the absence of glucose.^45^ In terms of autophagosome formation, Patrice Codogno et al confirmed that the ATGs are involved in the process through the ATG12-ATG5 and LC3-II complexes, then ATG12 binds to ATG7 and ATG10 to produce a ubiquitin-like reaction, then conjugates to ATG5, and finally reacts non-covalently with ATG16 to form a larger autophagosome complex.^46^ P62, also known as SQSTM1, serves as a selective autophagy bridging protein with multiple protein interaction domains capable of binding to Kelch-like ECH-associated protein 1 (Keap1). This binding may impair Keap1’s ability to degrade nuclear factor erythroid 2-related factor 2 (NRF2), thereby facilitating NRF2’s nuclear localization and the expression of downstream antioxidant genes. These changes may indirectly affect the process of autophagosome-lysosome fusion.^47^ Therefore, understanding the relevant molecules in the pathogenesis and development of autophagy is helpful to better control autophagy.

## The roles of ferroptosis and autophagy in diabetes

### Ferroptosis in diabetes

Recent research has demonstrated the critical role of iron as an antioxidant and trace element in various metabolic processes, including energy metabolism, oxygen transport, and other critical intracellular pathophysiological processes. Iron has also been shown to influence the etiology of diabetes. Emerging scientific evidence reveals that iron overload affects glucose metabolism, leading to hyperinsulinemia and insulin resistance, which is a known risk factor for type 2 diabetes mellitus. While in diabetes, protein glycosylation in turn stimulates transferrin to release iron, creating a vicious cycle.^48^ At the same time, there are research reports that iron overload also increases DMT1 expression in the pancreas, leading to insulin β-cells failure even in the absence of significant free iron deposition in the cells.^49^^,^^50^ Ionic iron chelators can inhibit ferroptosis by binding to free iron ions within cells, thereby improving conditions such as oxidative stress, cognitive dysfunction, neurovascular function, and kidney disease associated with diabetes.^51^, ^52^, ^53^, ^54^ In addition, iron chelation therapy to reduce iron overload has also been shown to lower blood sugar in type 2 diabetes mellitus.^55^ Notably, consequences from diabetes are also significantly influenced by ferroptosis brought on by iron metabolism. For instance, patients with hereditary hemochromatosis mutations have been found to have elevated iron levels in their lysosomal proximal renal tubules. Additionally, Fe^2+^ can stimulate the production of ROS through the Fenton reaction, thereby diminishing the antioxidant capacity of the cells and exacerbating diabetic kidney injury.^56^ Targeted activation of the HIF-1α/HO-1 pathway has the potential to cause iron mortality, damage to the renal tubules, and worsen diabetic nephropathy.^12^ Nonetheless, a high-concentration iron meal was found to reduce blood glucose and boost the number of anti-inflammatory M2 macrophages in a diabetic mouse model, indicating that diabetes may benefit from a specific amount of iron.^57^ Models of diabetic neurodegenerative illness show higher brain iron levels, which cause ferroptosis and cognitive dysfunction. On the other hand, better iron metabolism reduces beta amyloid (Aβ) aggregation and improves brain cognition.^58^^,^^59^ The precise relationship between iron supplementation and peripheral nerve inflammation and degeneration is still unknown, but some have hypothesized that iron supplementation may also aid in the recovery of diabetic foot ulcers based on the observed drop in serum iron levels in diabetic foot ulcers. Activation of the NRF2/FPN1 pathway may suppress ferroptosis to support the treatment of diabetic myocardial ischemia, in contrast to the observation of ferroptosis caused by a disruption in iron homeostasis in a diabetic rat model of myocardial ischemia.^60^^,^^61^ However, the potential confounding factors or methodological limitations in the aforementioned studies should be noted. To sum up, iron levels in the diabetic state must be strictly controlled because they may be a possible cofactor in diabetes. The roles of the various ferroptosis pathways mentioned above in diabetes and their molecular mechanisms are summarized in Table 1.

**Table 1:** Regulatory mechanisms of ferroptosis in diabetes mellitus.

Pathway	Possible mechanisms	Induce or inhibit ferroptosis	Diseases	References
Iron metabolism	Increased expression of DMT1 in the pancreas	Induce	Diabetes	45
	Promoting ROS production through Fenton reaction and reducing cellular antioxidant capacity	Induce	DN	53
	Activation of the HIF-1α/HO-1 pathway	Induce	DN	54
	Decreased blood glucose and increased number of anti-inflammatory M2 macrophages	Inhibit	Diabetes	55
	Increases oxidative stress	Induce	DNP	56,57
	Activation of the NRF2/FPN1 pathway	Induce	DCM	58,59
GSH/GPX4 pathway	Lower selenium levels reduce GPX4 synthesis	Induce	DFU	62
	Activation of p53-xCT-GSH axis	Induce	DCM	63
	Activated the NRF2/GPX4 pathway to attenuate oxidative stress	Inhibit	DCD, DCM	65,66
	Activation of KEAP1-NRF2-ARE antioxidant pathway and reduction of GPX4 depletion	Inhibit	DCM	68,69
	Reduced SLC7A11 expression decreases GPX4 synthesis	Induce	DKD	70
	Increased expression of GPX4 and SLC7A11	Inhibit	DKD	71
	Activation of the SIRT3-SOD2-GPX4 signaling pathway and maintenance of mitochondrial redox homeostasis	Inhibit	DKD	74
	Knockdown of DPEP1 expression attenuates GSH/GPX4 axis inhibition	Inhibit	DNP	75
	Poliumoside activates the NRF2/GPX4 signaling pathway	Inhibit	T2DOP	76
	Inhibit SLC7A11/GPX4 axis	Inhibit	DP	77
	ACSL4-LPCAT3-LOX is activated and iron overload and oxidative stress are reduced	Inhibit	DMED	77
	Inhibition of the YAP-GPX4 signaling pathway to attenuate oxidative stress	Inhibit	DR	78
Lipid metabolism	Reduced expression of antioxidant enzymes such as superoxide dismutase (SOD), peroxidase and catalase in glandular β cells	Induce	Diabetes	81,82
	Activation of the mitochondrial ROS-autophagy-lysosomal pathway leads to cellular ROS accumulation	Induce	Diabetes	85
	Activation of the endoplasmic reticulum (ER) stress response	Induce	Diabetes	86
Lipid metabolism	Targeting NRF2/HO-1/GPX4 and SLC7A11 significantly reduces ferritin levels	Inhibit	T2DOP	89
	Inhibition of peroxisome proliferator-activated receptor γ (PPARγ)-mediated iron death attenuates lipid peroxidation and oxidative stress	Inhibit	DR	90
	ROS buildup damages mitochondria	Induce	DR	91
	Reducing oxidative stress	Inhibit	DFU	92
	Increased ROS production further promotes oxidative stress	Induce	DCM	93
The NAD(P)H/FSP1/CoQ10 system	Ferroptosis suppressor protein 1 (FSP1) utilizes NAD(P)H to regenerate ubiquinol from its oxidized form	Induce	DFU	37
	Suppress lipid peroxidation	Induce	DFU	38
The GCH1/BH4/DHFR system	Insufficient availability of BH4 can lead to decoupling of nitric oxide synthases and the production of highly oxidative radicals	Induce	DNP	123
	Scavenges ROS and lipid peroxides within cells	Induce	DNP	123

**Notes:** DN, Diabetic Nephropathy; DKD, Diabetic Nephropathy; DNP, Diabetic Nephropathy; DP, Diabetic Polyneuropathy; DNP, Diabetic Neuropathy; DMED, Diabetic Male Erectile Dysfunction; DFU, Diabetic Foot Ulcer; DCM, Diabetic Cardiomyopathy; DR, Diabetic Retinopathy; DCD, Diabetic Chronic Kidney Disease; T2DOP, Type 2 Diabetes Osteoporosis.

GPX4 is an important intracellular antioxidant enzyme that uses glutathione to fight lipid peroxidation, protect many tissues, and maintain cell membrane stability.^62^ Since GPX4 is a selenoprotein, its proper operation may be jeopardized if cellular selenium levels are insufficient. It is worth noting, patients with diabetic foot ulcers had significantly decreased levels of selenium, which may decrease the synthesis of GPX4, raising the risk of diabetic foot ulcers and worsening wound healing.^63^ Remarkably, iron mortality in HUVEC is caused by activation of the p53-GSH-GPX4 axis by a high-glucose environment. The etiology of diabetic cardiovascular complications may include this as a key element.^19^ Ferroptosis has been demonstrated in the diabetic mouse brain. However, promoting NRF2/GPX4 to prevent ferroptosis may be able to prevent cognitively caused damage in the diabetic brain.^64^ Interestingly, stimulating the NRF2/GPX4 pathway could similarly decrease ferroptosis, hence lowering damage to diabetic cardiomyocytes.^65^^,^^66^ Concurrently, a different medication known as curcumin has the ability to decrease GPX4 depletion and activate the KEAP1-NRF2-ARE antioxidant response pathway, which prevents glucose-induced cardiomyocyte ferroptosis.^67^^,^^68^ Subsequently, it has been observed that SLC7A11 and GPX4 mRNA expression were indeed decreased in renal biopsy tissues of diabetic nephropathy patients, and targeting GPX4 may be an effective strategy for the treatment of diabetic nephropathy.^9^ Zinc-dependent metalloproteinase dipeptidyl peptidase 1 (DPEP1) has also been linked to ferroptosis in DN, and bioinformatics investigations have shown that it may speed up the GSH/GPX4 axis to prevent ferroptosis.^69^ Additionally, it has been discovered that the addition of the excellent antioxidant and anti-inflammatory compound chemosynthetic poliumoside can stimulate the NRF2/GPX4 signaling pathway to successfully inhibit ferroptosis and treat type 2 diabetic osteoporosis.^70^ It has been shown that diabetic periodontitis may inhibit the SLC7A11/GPX4 axis and trigger ferroptosis in alveolar osteoblasts, but treatment with resveratrol rescued this phenomenon.^71^ It has also been found that increasing GPX4 concentration by direct injection into the corpus cavernosum of diabetes-induced erectile dysfunction resulted in the activation of ACSL4-LPCAT3-LOX signaling while reducing iron overload and oxidative stress, suggesting it as a promising therapeutic target for preventing diabetes-induced erectile dysfunction.^72^ Clinical studies also found that the metabolite methylphenidate was significantly reduced in diabetic retinopathy and negatively correlated with blood glucose and glycosylated hemoglobin, and exogenous addition of methylphenidate could inhibit the YAP-GPX4 signaling pathway to alleviate oxidative stress and impede the progression of diabetic retinopathy.^73^ In conclusion, the GSH/GPX4 pathway can control one of the pathways behind diabetes pathogenesis, considerably expanding the range of potential treatments (Table 1).

Ferroptosis is known to be triggered by the accumulation of lipid peroxidation and ROS due to oxidative stress.^74^^,^^75^ Studies have demonstrated that long-term hyperglycemia induces endothelial oxidative stress and inflammation, resulting in lipid peroxidation accumulation. Iron overload exacerbates this effect, creating a detrimental cycle.^76^ Additionally, acterinol activates the endoplasmic reticulum stress response, causing insulin secretion dysfunction and inducing ferroptosis in mouse pancreatic β-cells.^77^ Conversely, quercetin, a natural polyphenol, shows promise in inhibiting ferroptosis in pancreatic cells by mitigating oxidative stress, thereby potentially exerting anti-diabetic effects.^78^ However, by initiating the downstream cellular oxidative defense processes of SOD and GSH, targeting NRF2/HO-1/GPX4 can significantly lower ferritin levels and attenuate the accumulation of lipid peroxides to attenuate the effects of type 2 diabetic osteoporosis.^79^ Recent studies have also demonstrated the impact of lipid peroxide-induced ferroptosis on diabetic complications, research directions, and potential therapeutic targets. For example, in high-glucose prone to lipid peroxidation, oxidative stress, and ferroptosis, inhibition of peroxisome proliferator-activated receptor γ-mediated ferroptosis can reduce lipid peroxidation and oxidative stress in diabetic retinopathy.^80^ In addition, clinical experiments have also found that ROS increases in diabetic patients, and ROS accumulation can damage mitochondria and further promote ROS production, forming a vicious circle that induces ferroptosis in capillary cells, ultimately leading to the occurrence of diabetic retinopathy.^81^ It is well established that persistent inflammation and oxidative stress at the wound site are the primary causes of chronic diabetic wounds. And targeting ferroptosis to mitigate oxidative stress represents a potentially novel therapeutic strategy for this condition.^82^ Studies have also found that diabetic cardiomyopathy is associated with ferroptosis by the mechanism that increased ROS production further promotes oxidative stress and damages cardiomyocytes.^83^ Interestingly, the study found that oxidative stress may be associated with insulin resistance, lipid metabolism disorders, and inflammation of neural tissues, which affects cognitive dysfunction in diabetic patients.^84^ Meanwhile, it was found that specific targeting of peroxisomal β-oxidation could inhibit high glucose-induced lipid accumulation and attenuate the damage in diabetic nephropathy.^85^ Ferroptosis has also been recently detected in animal models of diabetic nephropathy, leading to the hypothesis that reduction of oxidative stress, activation of the NRF2 antioxidant pathway, and control of inflammation may be more likely to modulate ferroptosis for diabetic nephropathy treatment purposes.^86^ In conclusion, attenuating lipid peroxidation or oxidative stress to modulate ferroptosis provides new ideas for the clinical management of diabetes (Table 1).

FSP1 inhibits ferroptosis by lowering the levels of coenzyme Q10, thereby halting lipid peroxidation. Research has shown that following the treatment of diabetic foot ulcers with vacuum sealing drainage, the expression of both FSP1 and CoQ10 increases in the tissue.^87^^,^^88^ Furthermore, it has also been discovered that the FSP1-CoQ10 pathway inhibits ferroptosis in renal tubular epithelial cells.^89^ Consequently, the application of FSP1 may be indispensable in future therapeutic strategies for both diabetic foot ulcers and diabetic nephropathy.

Research has demonstrated that increasing the expression of GTP cyclohydrolase 1 (GCH1) and the levels of BH4 can improve eNOS coupling. This reduction in excessive ROS in the aortas of diabetic mice and in human umbilical vein endothelial cells treated with high glucose serves to protect the endothelial function in diabetic mice.^90^

### Autophagy in diabetes

Many scientific studies have revealed that inhibition of mTORC1 is one of the effective ways to initiate activation of autophagy. It has been studied that mTORC1 is highly activated at high-glucose concentrations, inhibiting autophagy, and inhibiting mTORC1 can, in turn, enhance autophagy to improve pancreatic β cell function.^91^ Furthermore, Liu and Kang et al observed enhanced spinal cord autophagy activity by inhibiting the PI3K/AKT/mTORC1 signaling pathway, relieving neuropathic pain in diabetic rats.^92^ Studies have confirmed that insulin is a well-known inhibitor of autophagy, and its mechanism is that after insulin binds to cell surface receptors, on the one hand, it inhibits AMPK activity through IRS1 activation of PI3K-AKT pathway, and on the other hand, it activates mTORC1 activity through AKT-TSC1/2-RheB-mTORC1 pathway, and ultimately reduces diabetic autophagy activity.^93^^,^^94^ Metformin, a widely used antidiabetic drug, exerts its effects by enhancing autophagy activity through AMPK stimulation via ULK1 or by directly inhibiting mTORC1 phosphorylation. Moreover, AMPK can also promote autophagy in diabetic cardiomyopathy by activating beclin1, c-Jun N-terminal kinase 1 (JNK1), and the forkhead box transcription factor (FOXO).^95^ Similar to this, additional hypoglycemic drugs such as SGLT2 inhibitors, PPAR agonists, and GLP-1 agonists stimulate autophagy through the AMPK-TSC1/2-mTORC1 signaling pathway to lower blood sugar levels.^96^ Numerous AMPK activators have been shown to increase autophagy activity and alleviate diabetes problems, including omega-3 polyunsaturated fatty acids, metformin, cinacalcet, quercetin, resveratrol, progranulin, astragaloside IV, mangiferin, netrin, cinacalcet, and aspirin. Additionally, enhancing autophagy by inhibiting the PI3K-AKT-mTORC1 pathway will lessen the discomfort of diabetic neuropathy.^97^ Moreover, by modifying mTORC1/TFEB-mediated autophagy, TXNIP deficiency mitigates diabetic nephropathy.^98^ Specifically, the impaired autophagy brought on by diabetes heightens the development of diabetic cardiomyopathy by diminishing NRF2-mediated antioxidant defenses and inducing cardiomyocyte ferroptosis instead.^99^ TGF-β and mTOR pathway inhibition to promote autophagy slows the course of diabetes-induced erectile dysfunction.^100^ Through activating autophagy via the AMPK/mTORC1 signaling pathway, neuregulin-4, an adipokine protective against insulin resistance, attenuates diabetic cardiomyopathy.^101^ Therefore, modulating the expression of relevant molecules in the mTORC-dependent autophagy pathway presents a promising therapeutic approach for diabetes treatment (Table 2).

**Table 2:** Regulatory mechanisms of autophagy in diabetes mellitus.

Pathway	Possible mechanisms	Induce or inhibit autophagy	Diseases	References
The mTOR pathway	High glucose activates mTORC1 and inhibits autophagy, but inhibition of mTOR1 in turn enhances autophagy.	Induce	Diabetes	107
	Inhibits PI3K/AKT/mTOR signaling pathway	Induce	DNP	108
	Reduces autophagy by inhibiting AMPK or activating mTORC1 activity	Inhibit	Diabetes	109,110
	Stimulation of autophagy through the AMPK-TSC1/2-mTOR signaling pathway	Induce	Diabetes	112
	TXNIP deficiency regulates mTORC1/TFEB-mediated autophagy by	Inhibit	DKD	114
	Diabetes-induced autophagy defect reduces NRF2-mediated antioxidant defense and instead induces iron death	Inhibit	DCM	115
	Nrg4 can activate autophagy through activation of AMPK/mTOR signaling pathway	Induce	DCM	117
Other signaling pathways	Inhibition of Beclin1-dependent autophagy	Inhibit	DCM	118
	AMPK activation separates Bcl-2 from Beclin1, which induces autophagy	Inhibit	DCM	120
	Activation of endoplasmic reticulum stress and ASK1-JNK1/2 signaling	Induce	DNP	122
	JNK autophagy signaling pathway	Induce	Diabetes	123
	Inhibition of class III PI3K activity by 3-MA inhibits autophagy.	Inhibit	DNP	124
	DAPK3 further induces autophagy by activating ULK1	Induce	Diabetes	128

**Notes:** DKD, Diabetic Nephropathy; DNP, Diabetic Nephropathy; DCM, Diabetic Cardiomyopathy.

Beclin1, a significant ATG protein, acts as a recognized regulator and contributes to the formation of the PtdIns3K complexes that activate autophagy. Studies have demonstrated that elderly diabetic mice are susceptible to cognitive and emotional impairment, which is associated with Beclin1-mediated autophagy.^102^ In the context of acute hyperglycemia-induced myocardial damage, sodium-glucose cotransporter type 2 inhibitors can effectively ameliorate myocardial injury in nondiabetic myocardial infarction combined with acute hyperglycemia by inhibiting beclin1-dependent autophagy.^103^ Furthermore, literature reports indicate that AMPK activation can dissociate Bcl-2 from Beclin1, leading to autophagy induction and potentially preventing diabetic cardiomyopathy.^104^ Taken together, targeting the key autophagy regulator Beclin1 may hold promise as a therapeutic approach for diabetes.

By participating in activities like endoplasmic reticulum stress, controlling the expression of numerous autophagy-related genes through FOXO-dependent transcription, facilitating post-translational modification of Bcl-2, and mediating the detachment of beclin1 and Bcl-2,^105^ the MAPK-JNK signaling cascade had a direct impact on autophagy. Endoplasmic reticulum stress and ASK1-JNK1/2 signaling have been theorized as potential contributors to cognitive dysfunction in diabetes.^106^ Additionally, the loss of the glucose-reactive protein GRP78 causes endoplasmic reticulum stress, which in some cases results in JNK activation, may help protect the function of β cells and thus prevent diabetes.^107^ Consequently, altering the JNK autophagy signaling pathway is a viable option for both treating and preventing diabetes.

Additional autophagy signaling pathways that have received less attention are also closely associated with diabetes. For instance, research has shown that the substance 3-methyladenine (3-MA) can block autophagy and, to a certain extent, halt the progression of diabetes by inhibiting class III PI3K activity. Moreover, 3-MA-induced autophagy inhibition exacerbates cognitive impairment in streptozotocin-induced diabetic mice.^108^ Furthermore, inadvertent cell death of β cells can occur when autophagy is inhibited by 3-MA.^109^ Conversely, aberrant expression of autophagy-related proteins aggravates brain damage in diabetic patients, but 3-MA application can attenuate severe brain damage and restore its function. Therefore, autophagy modulation might be a potential therapeutic strategy for diabetes.^110^

Death-associated protein kinase 3 (DAPK3), which can control a variety of cellular processes, plays another role in the regulation of autophagy. Without altering the expression of autophagy genes at normal glucose settings, DAPK3 knockdown can further induce autophagy by activating ULK1 under high-glucose conditions.^111^ Similarly, suppression or absence of Drak2, a type of DAPK, may encourage the protection of cells against apoptosis. Consequently, Drak2 might be a useful target for the creation of relevant hypoglycemic drugs.^112^ Overall, a thorough investigation of the etiology of autophagy in diabetes offers scientific guidance for comprehending, researching, developing, and treating diabetes and its associated problems. Table 2 provides a summary of the aforementioned autophagy pathways' functions in diabetes as well as their molecular underpinnings.

Notably, autophagy and ferroptosis play distinct roles in different diabetic complications. In diabetic cardiomyopathy, autophagy often exhibits a dual role. In early stages, it removes damaged mitochondria to reduce oxidative stress, thereby exerting a protective effect. However, in later stages, excessive autophagy may degrade functional proteins and organelles in cardiomyocytes, aggravating myocardial damage.^113^ In contrast, ferroptosis is mainly driven by the downregulation of GPX4 and SLC7A11, leading to iron accumulation and lipid peroxidation, which directly promote cardiomyocyte death and myocardial fibrosis.^114^

In diabetic nephropathy, autophagy is more inclined to play a protective role in the early stage by clearing damaged renal tubular epithelial cells and reducing the accumulation of extracellular matrix. But with the progression of the disease, the autophagic capacity of renal cells decreases, which cannot effectively remove harmful substances, thus promoting the development of renal interstitial fibrosis.^115^ Ferroptosis is more prominent in renal tubular epithelial cells. High-glucose environment induces the increase of transferrin receptor 1, which enhances iron uptake, and meanwhile, the decrease of GPX4 activity makes the cells unable to resist lipid peroxidation, leading to renal tubular damage.^116^

In diabetic retinopathy, autophagy is mainly involved in the regulation of retinal vascular endothelial cells. Moderate autophagy can maintain the stability of vascular endothelial cells, while excessive autophagy may lead to endothelial cell death and promote the formation of retinal neovascularization.^117^ Ferroptosis here is closely related to the damage of retinal pigment epithelial cells. The decrease of antioxidant capacity and the disorder of iron metabolism in these cells lead to lipid peroxidation, which affects the function of the retinal barrier and accelerates the progression of retinopathy.^118^

### Interaction mechanisms between autophagy and ferroptosis in diabetes

Recent studies increasingly demonstrate that ferroptosis is autophagy-dependent, relying on autophagic processes to facilitate iron ion accumulation and lipid peroxidation. When autophagy is overactivated or lysosomal activity is dysregulated, these pathways synergistically promote cell death. Given the pivotal roles of autophagy and ferroptosis in diabetes, elucidating their interplay and downstream effects has emerged as a critical research focus for developing novel anti-diabetic therapies.

### Autophagy and ferroptosis interact through iron metabolism pathways

Ferroptosis is influenced by autophagy, which controls iron ion metabolism. Ferritinophagy is an NCOA4-mediated process for the selective autophagic breakdown of ferritin. Notably, ferritinophagy flux is mostly determined by the amount of NCOA4. Along with this process, autophagosome LC3 and lysosomes fuse to form autophagosomes, and ferritin finally breaks down to liberate free iron.^119^ Consequently, autophagy’s role in ferroptosis is functionally dependent. Ferritinophagy, which is closely linked to diabetes and its complications, involves NCOA4, a key driver of ferritin catabolism. Notably, diabetic mice exhibit reduced ferritin levels and elevated NCOA4 expression in neuronal cells, indicating that high-glucose environments induce ferritinophagy. Intriguingly, melatonin administration ameliorates neuronal damage by inhibiting NCOA4-mediated ferritinophagy through miR-214-3p inhibition.^120^ In addition, the long-term accumulation of senescent cells in the diabetic environment makes the cells insensitive to ferroptosis, and the elimination of senescent cells in diabetic wounds by using AMPK to activate NCOA4-mediated ferritinophagy can promote wound healing.^121^ However, during diabetic myocardial ischemia/reperfusion injury, the levels of both DNMT-1 and NCOA4 were increased, but inhibition of DNMT-1 expression could, in turn, reduce NCOA4-mediated ferritinophagy to mitigate myocardial injury.^122^ Similar to this, the protein mitochondrial ferritin (FtMt), which stores ferric ions and intercepts toxic ferrous ions in cellular mitochondria, can inhibit ferroptosis in osteoblasts by reducing oxidative stress caused by excess ferrous ions. Additionally, a deficiency in FtMt induces mitochondrial autophagy via the ROS/PINK1/Parkin pathway, thereby making FtMt a potential target for type 2 diabetic osteoporosis therapy.^123^ In essence, autophagy and functional lysosomes can influence iron metabolism and thereby promote ferroptosis. This pathway holds potential as a novel avenue for treating complications associated with diabetes, such as cardiovascular disease, nephropathy, neuropathy, and infection.^124^ The role of ferritinophagy in diabetes and its consequences remains largely unexplored. Considering the connection between autophagy-mediated iron metabolism regulation and ferroptosis, future studies investigating the NCOA4-mediated ferritinophagy pathway and its involvement in diabetes-related complications will be highly valuable.

### Autophagy and ferroptosis interact through the antioxidant system

Ferroptosis and autophagy are both induced by GSH depletion, linking these pathways in the pathophysiological processes of diabetes. Studies have demonstrated that autophagy inhibitors, such as Baf-A1 and 3-MA, can prevent GSH depletion-induced ferroptosis.^125^ Additionally, in the type 2 diabetic osteoporosis model, high-glucose environments have been shown to concentration-dependently induce ferroptosis while simultaneously upregulating the expression of acidic sphingomyelinase (ASM) and autophagy. On the other hand, ASM inhibition improves osteogenic function by reducing high glucose-induced autophagy, GPX4 degradation, and subsequent ferroptosis. In this scenario, ASM-mediated autophagic activation plays a critical role in promoting GPX4 degradation induced by high-glucose levels.^126^ Moreover, through chaperone-mediated autophagy, the heat shock protein 90 controls the lysosome-associated membrane protein-2a metabolism. This modulation accelerates GPX4 degradation and activates ferroptosis.^127^ It has also been shown that Sirtuin 3 (SIRT3) expression is significantly elevated in high-glucose environments, and that SIRT3 induces ferroptosis and autophagy in trophoblast cells by promoting the AMPK-mTOR pathway and decreasing the level of GPX4, a process that is involved in the pathogenesis of gestational diabetes mellitus.^128^ According to these results, GSH seems to be involved in both ferroptosis and autophagy. However, further research is needed to determine the precise processes by which GSH functions in diabetes mellitus.

CoQ10 treatment has been found to alleviate neuronal damage, promote mitochondrial autophagy, and improve cognitive impairments in mice.^129^ Additionally, it can activate the PI3K/AKT/mTOR signaling pathway to inhibit autophagy, thereby suppressing the activation of pancreatic stellate cells and ameliorating pancreatitis.^130^ This may represent a promising alternative therapeutic approach for diabetes in the future. The anti-apoptotic proteins Bcl-2, Bcl-xL, and Mcl-1, which share the BH1–BH4 structural domains, can dynamically regulate autophagy through their interaction with Beclin-1, playing crucial roles in numerous diseases.^131^

### Autophagy and ferroptosis interact through the lipid metabolism pathways

Autophagy exerts its influence on ferroptosis by modulating lipid metabolism pathways. Endoplasmic reticulum stress, ferroptosis, and autophagy are all well-known to be impacted by ROS. By triggering endoplasmic reticulum stress and autophagy, cadmium can harm tubular epithelial cells, and the production of ROS during this process may contribute. Animal models of type 2 diabetes have been found to have increased levels of lipid peroxides, including 4-HNE, MDA, etc. These lipid peroxides can obstruct glucose absorption and directly harm pre-adipocyte insulin signaling.^132^ To stop lipid peroxides from building up in insulin-sensitive peripheral tissues, autophagy is essential.^133^ High-glucose levels also significantly contribute to oxidative stress-induced ferroptosis,^134^ and blocking endoplasmic reticulum stress-induced autophagy-mediated ferroptosis can, on the other hand, increase angiogenesis.^135^ Moreover, autophagy was found to play a dual role in diabetic cardiomyopathy. For one thing, in chronic diabetes, autophagy defects lead to the loss of NRF2-mediated redox defense mechanisms, and damage to autophagy proteins leads to the accumulation of ROS, which further impairs cardiac function, a process that has the potential to induce ferroptosis. For the other thing, autophagy can, in turn, activate NRF2 to enhance the antioxidant capacity of cells to play a protective role,^136^^,^^137^ and this may also accelerate ferroptosis. The pathophysiology of diabetes and its consequences may be influenced by endoplasmic reticulum stress and ROS, and the lipid metabolism route may connect iron-dependent cell death and autophagy in diabetes.

### Autophagy and ferroptosis interact through their modulation of various regulatory proteins

**Figure 2:**
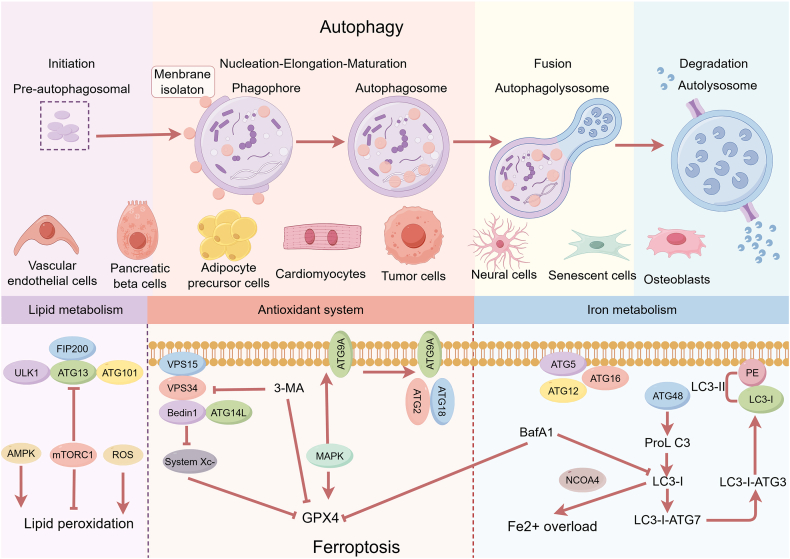
Mechanisms of autophagy and ferroptosis in diabetic complications. The process of autophagy is divided into five different stages: initiation, vesicle nucleation, vesicle elongation, vesicle fusion, and cargo degradation. Each stage can affect ferroptosis by influencing iron metabolism, GPX4 expression, and the lipid peroxidation pathway. The figure was generated with Figure Draw.

A growing body of research has found that coregulators such as Beclin1, NRF2, p53, JNK, and AMPK play a crucial role in ferroptosis and autophagy in diabetes and its complications (Fig. 2). For instance, studies have revealed that the autophagosome initiator protein Beclin1, upon activation by AMPK phosphorylation, can bind to SLC7A11, an inhibitor of system Xc^–^. This interaction reduces the activity of system Xc^–^, contributing to ferroptosis and enhancing autophagy.^125^^,^^138^ Additionally, Beclin1 can bind to the outer capsule of adipose tissue, promoting adiponectin secretion and improving insulin sensitivity.^139^ Inhibition of Beclin1 expression reduces the inhibition of system Xc^–^ activity by iron-dependent cell death inducers like Erastin.^125^

p53 has also been found to play a unique role in different studies of ferroptosis and autophagy. For example, the p53-AMPK-mTORC1 signaling pathway can influence SLC7A11 expression and ROS buildup to enhance iron-dependent cell death while either promoting or inhibiting autophagy.^127^^,^^140^ Increased p53 protein levels caused by hyperglycemia can result in diabetic hearts experiencing loss of capillary density.^141^ Dysregulation of autophagy in diabetic nephropathy may be associated with the p53/miR-214/ULK1 axis and could serve as a therapeutic target for diabetic nephropathy.^140^

NRF2 plays a crucial role in ferroptosis by targeting and regulating system Xc^–^ activity and GPX4 expression. In addition to upregulating autophagy-related genes, NRF2 activation can also influence ferroptosis.^142^^,^^143^ In diabetic myocardial injury, autophagy is inhibited, but NRF2-mediated iron-dependent cell death pathways are activated, exacerbating disease progression.^99^ Notably, impaired autophagy triggers NRF2-induced ROS accumulation, promoting cellular necrosis.

Inhibiting the IRE1/JNK pathway can prevent ferroptosis in renal tubular epithelial cells; the JNK signaling pathway is involved in the regulation of autophagy,^105^ and renal tubular epithelial cells can stop ferroptosis by blocking the IRE1/JNK pathway.^144^ Under hypoxic conditions, JNK can also suppress NCOA4 transcription, which inhibits ferritin autophagy and ferroptosis.^145^

By controlling phosphorylated autophagy proteins and stimulating the mTORC1-ULK1 axis, AMPK can promote autophagy. Metformin, an AMPK activator, inhibits mTORC1 and increases autophagy activity, thereby lowering blood sugar levels.^146^ In conclusion, future research and the creation of diabetic therapeutics can benefit greatly from understanding and targeting these regulators.

## Conclusions

In recent years, “cell death” therapies, particularly those targeting ferroptosis and autophagy, have gained wide attention in diabetes research. Both processes play key roles in diabetes pathogenesis and hold promise as therapeutic targets for diabetes and its complications. Unlike conventional surgical or oral medications, modulating ferroptosis and autophagy at the cellular and molecular levels offers a more precise approach to treatment. Intriguingly, emerging evidence suggests that autophagy and ferroptosis can interact synergistically or antagonistically, depending on context and timing. This dynamic interplay highlights their potential as combined therapeutic strategies for diabetes management.

Clinical translational research on ferroptosis and autophagy is an emerging topic and a critical yet challenging task, involving exploration of their shared interactions in diabetes treatment. In this review, we also focus on the possible interactions between autophagy and ferroptosis in diabetes mellitus and their consequences. Studies have generally shown that iron-dependent cell death is autophagy-dependent and that autophagy interacts with iron-dependent cell death through three major regulatory pathways, namely, iron metabolism, antioxidant system, and lipid metabolism. Synergistic regulators, such as Beclin1, NRF2, p53, JNK, and AMPK, serve as a bridge between autophagy and ferroptosis. Interestingly, although autophagy can influence ferroptosis in diabetes through the above pathways, ferroptosis also, in turn, inhibits or activates autophagy, forming a kind of closed loop. This suggests a potential dual role for ferroptosis and autophagy. Future studies could aim to elucidate this emerging area of research. However, caution is warranted here because such targeted therapeutic strategies against ferroptosis and autophagy may be a double-edged sword. For example, while activation of autophagy may induce ferroptosis in diabetic wounds to remove senescent cells and promote wound healing, overactivation of autophagy may also exacerbate the development of diabetes by triggering a decline in insulin function. Autophagy exhibits distinct regulatory effects in different types of diabetes. In type 2 diabetes mellitus, rapamycin-induced autophagy activation has been shown to ameliorate DN, whereas it may exacerbate renal pathological damage in type 1 diabetes mellitus models.^147^ Importantly, excessive autophagy activation may induce cardiomyocyte damage, thereby exacerbating the progression of diabetic cardiomyopathy,^148^ while concurrently promoting skeletal muscle atrophy associated with DN.^149^ Intriguingly, in models of diabetic liver injury, autophagy demonstrates a protective role against ferroptosis-mediated hepatocyte damage through regulation of ACSL4 protein degradation.^150^ Nonetheless, the combination of autophagy and ferroptosis has been demonstrated to play an important role in diabetes genesis and may preserve the therapeutic efficacy of the treatment of diabetes. In future studies, targeted therapies aimed at utilizing ferroptosis and autophagy require more comprehensive safety assessments, and strategies to mitigate potential adverse effects should be actively explored.

The regulation of autophagy and ferroptosis in diabetes treatment presents both opportunities and challenges. Major challenges include: i) Precise modulation of the “double-edged sword” effect of autophagy to balance β-cell protection and complication management; ii) Insufficient specificity in ferroptosis-targeted interventions, which may disrupt normal iron metabolism; iii) Unclear dynamic mechanisms of their crosstalk in diabetes. Future research should focus on: i) applying multi-omics technologies to elucidate key regulatory nodes; ii) developing microenvironment-responsive smart drugs and targeted delivery systems; iii) conducting stratified clinical studies to validate personalized treatment regimens. These breakthroughs will advance precision medicine in diabetes therapy.

## CRediT authorship contribution statement

**Erlian Xie:** Writing – review & editing, Writing – original draft. **Xuerong Wei:** Visualization, Validation. **Zijun Zheng:** Supervision, Software. **Qiuyi Yu:** Resources, Project administration, Conceptualization. **Mengqian Liu:** Funding acquisition, Formal analysis. **Huihui Zhang:** Data curation. **Ziwei Jiang:** Conceptualization. **Yanbin Gao:** Data curation. **Lei Yang:** Funding acquisition.

## Funding

This work was financially supported by the 10.13039/501100001809National Natural Science Foundation of China (No. 82372526, 82302797) and 10.13039/501100021171Guangdong Basic and Applied Basic Research Foundation (China) (No. 2023A1515012970, 306217527025).

## Conflict of interests

The authors declared no competing interests.
